# 

**DOI:** 10.1192/bjb.2022.58

**Published:** 2023-06

**Authors:** Rebecca Lawrence

**Affiliations:** is a consultant psychiatrist with NHS Lothian, Edinburgh, UK. Email: rebecca.lawrence@nhslothian.scot.nhs.uk

**Figure:**
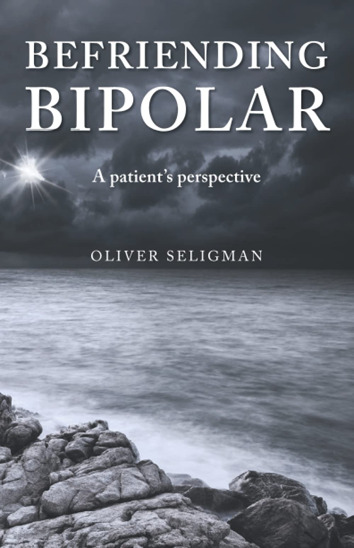

*Befriending Bipolar* is a curious title for a book written by someone whose life has been profoundly affected by this illness and its treatment. Oliver Seligman's searing descriptions of his experiences are, at times, shocking, but there is a gentleness in his approach that also renders them apt.

He was very young when he first became ill, and perhaps the one advantage of the severity of his illness was that he was rapidly diagnosed, after a difficult stay in hospital that can only hint at his future psychotic episodes. Perhaps more importantly, one of his earliest psychiatrists is someone who listens to him and allows him time, before supporting him to start lithium, the one medication that seems to keep him well enough to live. It is not without its problems and side-effects, though, and this is a prevailing theme throughout the book.

Seligman gives eloquent descriptions of the different states of mind he has experienced, ranging from extreme mania to severe depression, frequently punctuated by florid psychosis. But he also talks about the times between episodes, and how difficult and unclear these can be – even when seemingly well, bipolar disorder affects the whole fabric of his life. It is moving to read of his regrets and guilt, particularly in relation to the effects of his illness on his wife and family. He also incidentally mentions being rejected by medical schools despite excellent grades, which, as a doctor, made me feel angry on his behalf; I better understood his own acceptance of the consequences of his illness as I read on.

I would strongly recommend this book to all psychiatrists. Seligman writes of the importance of being heard and also about the need for time and love, as well as medication and hospital admission when necessary. I was drawn to his recognition that he was only helped by a happy psychologist; this could probably be extended to psychiatrists and other professionals.

This is a story of acceptance and understanding, and, despite years of travails and illness, dominated by attempts to come off lithium, Seligman achieves a balance by taking a lower dose. But he also identifies many other things that help him, including humour, sleep and friendships, as well as meditation, which he has practised for 20 years, although interestingly not when unwell. It is a wonderful patient's perspective and, by the end, the title made perfect sense.

